# The value of (pre)school playgrounds for children’s physical activity level: a systematic review

**DOI:** 10.1186/1479-5868-11-59

**Published:** 2014-05-03

**Authors:** Karen Broekhuizen, Anne-Marie Scholten, Sanne I de Vries

**Affiliations:** 1 TNO, Department of Life Style, P.O. Box 2215, 2301 CE Leiden, Netherlands; 2 Institute for Evidence-Based Medicine in Old Age IEMO, P.O. Box 9600, 2300 RC Leiden, Netherlands; 3 The Hague University of Applied Sciences, Nutrition and Dietetics, P.O. Box 13336, 2501 EH The Hague, Netherlands; 4 Research group Healthy Lifestyle in a Supporting Environment, The Hague University of Applied Sciences, P.O. Box 13336, 2501 EH The Hague, Netherlands

**Keywords:** Playground, Physical activity, Children, Kindergarten, Schoolyard, Recess

## Abstract

The (pre)school environment is an important setting to improve children’s health. Especially, the (pre)school playground provides a major opportunity to intervene. This review presents an overview of the existing evidence on the value of both school and preschool playgrounds on children’s health in terms of physical activity, cognitive and social outcomes. In addition, we aimed to identify which playground characteristics are the strongest correlates of beneficial effects and for which subgroups of children effects are most distinct. In total, 13 experimental and 17 observational studies have been summarized of which 10 (77%) and 16 (94%) demonstrated moderate to high methodological quality, respectively. Nearly all experimental studies (n = 11) evaluated intervention effects on time spent in different levels of physical activity during recess. Research on the effects of (pre)school playgrounds on cognitive and social outcomes is scarce (n = 2). The experimental studies generated moderate evidence for an effect of the provision of play equipment, inconclusive evidence for an effect of the use of playground markings, allocating play space and for multi-component interventions, and no evidence for an effect of decreasing playground density, the promotion of physical activity by staff and increasing recess duration on children’s health. In line with this, observational studies showed positive associations between play equipment and children’s physical activity level. In contrast to experimental studies, significant associations were also found between children’s physical activity and a decreased playground density and increased recess duration. To confirm the findings of this review, researchers are advised to conduct more experimental studies with a randomized controlled design and to incorporate the assessment of implementation strategies and process evaluations to reveal which intervention strategies and playground characteristics are most effective.

## Introduction

During childhood and adolescence, regular physical activity is associated with improvements in both physiological and psychological health [1-4]. Despite the growing awareness of these benefits, children’s physical activity level seems to be declining [5,6]. Several studies have shown that many children are currently insufficiently active and do not meet the health-related physical activity guideline of ‘180 minutes or more of physical activity at any intensity spread throughout the day’ for preschool children [7,8] or the guideline of ‘60 minutes or more of at least moderate intensity activity each day’ for school-aged children [9]. Therefore, the promotion of regular physical activity in youth has become a public health priority.

Physical activity is influenced by many factors. Several reviews have summarized the evidence on correlates of children’s physical activity [10-15]. There is extensive literature on the demographic, biological, and psychosocial determinants of physical activity among youth [10,13-15]. In the last decade, a growing interest in the role of the built environment on physical activity can be observed. Ferreira et al. [11] conducted a semi-quantitative review of 150 studies on environmental correlates of youth physical activity published between 1980 and 2004, and found that particularly the school environment is associated with children’s physical activity level. This was subscribed in a similar, but smaller review of Davison & Lawson [12]. School is known as a suitable setting for the promotion of physical activity in youth, since children can be reached with minimal effort and children spent most of their time there [16]. School-based opportunities to engage in physical activity are during physical education classes, during recess and after school hours [17,18]. In contrast to physical education, which only provides 8% to 11% of children’s daily physical activity on a weekday [19,20], recess offers the potential to gather up to 40% of the daily amount of moderate to vigorous physical activity (MVPA) [21,22]. In 2006, Ridgers et al. summarized the effects of the first recess-based interventions and showed that energy expenditure and physical activity levels of children aged 4 to 12 years old increased shortly after the implementation of playground-based interventions at schools [22]. However, not all intervention strategies seem to be as effective [23-25]. According to Escalante et al. [25] who summarized five experimental studies, interventions based on playground markings, game equipment, or a combination of the two do not increase the physical activity level of children aged 4–11 years, but interventions based on playgrounds markings plus physical structures can be effective in the short to medium term. This is in contrast with Parrish et al. who summarized six experimental studies [24]. In their review, they state that playgrounds markings and games equipment significantly increase children’s physical activity level. Studies that examined combined strategies showed mixed findings. They conclude that although there are some promising recess-based interventions, there is no conclusive evidence for an effect of any type of recess-based intervention on the physical activity level of children aged 5–11 years. So far, none of the reviews have examined the effects of playground-based interventions on preschool children. Preschool children may also benefit from playgrounds [15]. In addition, none of the reviews have looked for evidence on the beneficial effects of playgrounds on outcomes other than physical activity.

Therefore, the aim of this review paper is to present an overview of the existing evidence on the value of (pre)school playgrounds for children’s health in terms of physical activity, cognitive and social outcomes. Further, we aimed to identify which playground characteristics are most effective, and for which subgroups of children effects are most distinct. In contrast to previous reviews on this topic [22-25], both observational and experimental studies focusing on preschool children as well as older age groups will be included, allowing for comparison of the results.

## Methods

### Search strategy and data sources

Studies published from January 2000 to September 2012 were identified through a structured computerized search of PubMed, PsycINFO, and EMBASE. The search terms are shown per database in Additional file 1. According to the search terms, only studies conducted in children from 2 to 18 years old were selected. In addition to these terms, related and most recent thesaurus terms of the search engines were added. No limitations for study design were added. Based on the title, search results were checked for relevance and duplicates.

### Selection of studies

Based on the title and the abstract, further study selection was performed by two independent researchers (KB and AMS). Studies had to examine the association between a (pre)school playground and physical, cognitive or social outcomes. (Pre)school playgrounds were defined as spaces located on (pre)school properties that were specifically designed for outdoor play and sports activities for children from 2 to 18 years old. Studies on other playgrounds, e.g., amusement parks or recreation areas that were not school-based were not included. Further, studies were included if published in a peer reviewed scientific journal and published in English. In addition, grey literature from January 2000 to September 2012 was identified through the Educational Resources Information Centre (ERIC) and the Dutch database ‘Grey Literature in the Netherlands’ (GLIN) using the search terms ‘schoolyard’ and ‘playground’. Grey literature formed a contextual background for the interpretation of the topic and results. Data from grey literature was not further extracted.

### Data extraction

Based on the full-texts of the studies, the data of each study was extracted by two independent researchers (KB and AMS or KB and SdV). In case of disagreement, this was discussed until consensus was reached. The following study characteristics were extracted: design of the study, level of randomization, aim of the study, size and source of the study sample, country in which the study was performed, age range and/or mean age of the sample, socio-economic status of the sample, type of playground and characteristics, type of outcomes, measurement instruments of playgrounds and outcomes, and effects per outcome. If available, additional results per subgroup (e.g., according to sex) were extracted.

### Methodological quality

Methodological quality was assessed by two independent researchers (KB and AMS or KB and SdV), based on the full-texts of the studies. Two scoring lists were developed for observational and experimental studies respectively. Items were derived from scoring lists of Prins et al. [26] and De Vries et al. [27]. The scoring list for observational studies contained 11 items: five items that indicated internal validity (reported validity and reliability of measurement instruments of the playground, reported validity and reliability of the outcomes, and report of statistical analytical procedure) and six items that indicated external validity (representativeness of the study sample, specification of the age range of the study sample, specification of in- and exclusion criteria, response rate or specification of non-response, specification of the study period, and specification of the sample characteristics).

The scoring list for experimental studies contained 14 items: nine items identical to those scored for observational studies, except for the reported validity and reliability of measurement instruments of the playground. Five items were specific for experimental studies, i.e., presence of a control group, randomization, blinding of study participants and interventionists, blinding of outcome assessors, and completeness of outcome data.

Each item was scored with ‘present’ (1), ‘partly present’ (0.5), or ‘absent’ (0), in accordance with De Vries et al. [27]. A total score was computed per publication by summing all unweighted scores. Each publication was then assigned a methodological quality rating. For experimental studies, methodological quality was high if 10 points or more were assigned, indicating that 72% of the quality criteria were met. The methodological quality of experimental studies was moderate if 7.0 – 9.5 points were assigned and low if 6.5 or less points were assigned. For observational studies, methodological quality was high if 8.5 points or more were assigned, indicating that 77% of the quality criteria were met. The methodological quality of observational studies was moderate if 5.5 – 8.0 points were assigned and low if 5.0 or less points were assigned. In case of disagreement between the two independent researchers, this was discussed until consensus was reached.

### Data synthesis

#### Level of evidence for playground-based intervention strategies

In order to summarize the level of evidence of the findings from experimental studies, intervention strategies used in the experimental studies were labelled with a level of evidence, ranging from strong, moderate, limited, inconclusive to no evidence. This rating system was used in previous reviews of Van Sluijs et al. [16] and Parrish et al. [24] and takes into account: study design, sample size, methodological quality, and the intervention effect. The decision-making process underlying the rating system is available as a supplementary file by Van Sluijs and colleagues [16]. In short, intervention strategies were labelled with strong, moderate or limited level of evidence if at least two-third of the studies found significant positive results. In order to be labelled as a large study, more than 250 participants were required.

#### Associations of playground characteristics with physical activity

The results of the observational studies were summarized in a slightly different manner. For each playground characteristic a summary code was determined taking into account the outcome, the methodological quality of the studies, and the total number of studies that examined the playground characteristic. Playground characteristics were categorized as either hardware (i.e., permanent playground conditions, such as playground size, and surface type), software (i.e., the provided equipment and activities on playgrounds, such as fixed and portable play equipment), or orgware (i.e., the organization beyond playgrounds, such as the presence of supervision and the recess duration). First, all outcomes were weighted for the quality of the study as previously done by De Vries et al. [27]. Observational studies of poor quality (≤5.0 points) provide less evidence for the reported associations than studies of high quality (≥8.5 points). Next, the number of studies that found significant positive or negative associations between the playground characteristic and physical activity was divided by the total number of studies that examined that characteristic. This resulted in a percentage of studies that supported a significant positive or negative association with physical activity. This percentage was classified as no association (0), indeterminate/inconclusive association (?), positive (+) or negative association (−) using the model of Sallis [13]. When 0%-33% of the studies supported a positive or negative association, the result was classified as no association (0). An indeterminate/ inconclusive (?) classification was determined if 34%-59% of the studies supported an association. A positive (+) or negative (−) association was determined when 60%-100% of the studies supported the direction of the association. When the quality of the underlying studies was moderate or high one or two additional characters (− or +) were assigned to the summary code, respectively. In addition, if a playground characteristic was investigated four or more times an additional + or – was also assigned [13].

## Results

### Selection of studies

**Figure 1:**
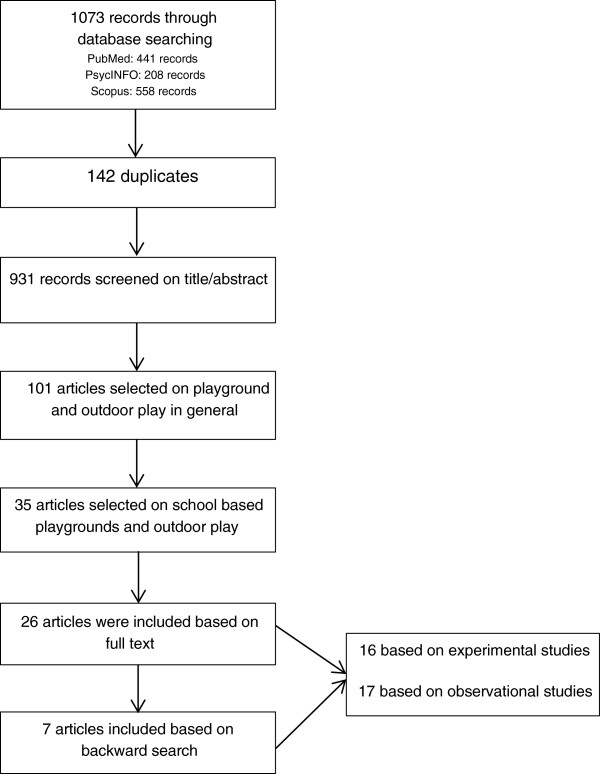
Flowchart describing the number of articles retrieved, and included and excluded at each stage of the review process.

The initial cross-database search in PubMed, PsycINFO, and EMBASE resulted in 1073 publications. After eliminating duplicates, 931 publications remained. Titles and abstracts were reviewed for eligibility criteria, resulting in 35 publications that were fully considered. Based on the full-texts, 26 of them were included in the review. A backward search of the reference lists of these publications yielded another seven publications. Thus, 33 publications were finally included. A flowchart of the selection procedure is depicted in Figure 1.

### Study characteristics

Of the 33 included publications, 16 reported on experimental studies and 17 on observational studies. Their study characteristics are summarized in Table 1 and Table 2, respectively.

**Table 1:** Characteristics of experimental studies (n = 13) and effects of (pre)school playgrounds on children’s health

**Study;**	**Study design;**	**School(s) (type)**	**Outcome (unit) [measurement]**	**Intervention effects**
**Country;**	**Level of randomization;**	**Intervention mode(s)**		
**Methodological quality**	**Study population (mean age/range; % girls)**			
**PRESCHOOL INTERVENTIONS**				
Brown, 2009 [37,47]	Non-randomized controlled trial	2 preschools	MVPA (% of intervals in which MVPA is performed) [OSRAP]^1^	No significant difference on intervention days compared to no-intervention days
US	5 children (80% girls)	Teacher-implemented promotion of MVPA (3 children)		
5.5		Teacher-implemented promotion of MVPA + guided discussions, initial pep talks on the playground, teacher participation, brief review and acknowledgement after the activity, and stickers for child participation (2 children)		
		No-intervention days (5 children)		
Cardon, 2009 [33]	RCT	40 preschools	I. % in sedentary activity during recess	I-V. No significant differences in intervention schools compared to control schools
Belgium	Randomization: school-level	Provision of play equipment (10 schools)	II. % in LPA during recess	
10	583 children (mean age 5.3; 47% girls)	Painting of playground markings (10 schools)	III. % in MPA during recess	
		Provision of play equipment *and* painting of playground markings (10 schools)	IV. % in VPA during recess	
		No intervention (10 schools)	V. % in average PA during recess[accelerometer]	
Hannon, 2008 [36]	Non-randomized trial 64 children (age 3–5; 53% girls)	1 preschool	I. % time spent in sedentary activity	I. Significant decrease after the intervention compared to pre-intervention *(F(1,61) = 243.90)*^*a*^
US		Provision of play equipment: hurdles to jump over and hoops to jump through, tunnels to crawl through, balance beams, target toss/throw sets, bean bags, various sized playground balls	II. % time spent in LPA	II. Significant increase after the intervention compared to pre-intervention *(F(1,61) = 16.30)*^*a*^
9			III. % time spent in MPA	III. Significant increase after the intervention compared to pre-intervention *(F(1,61) = 212.43)*^*a*^
			IV. % time spent in VPA [accelerometer]	IV. Significant increase after the intervention compared to pre-intervention *(F(1,61) = 50.35)*^*a*^
				Secondary analyses:
				Younger children showed significantly more moderate activity after the intervention compared to pre-intervention than older children *(F(2,61) = 9.64)*^*a*^
				Older children showed more vigorous activity after the intervention compared to pre-intervention than younger children *(F(2,61) = 2.83)*^*a*^
Holmes, 2006 [38]	Non-randomized trial	1 preschool	Post-recess attention (% attentive) [observations]	Significant increase in post-recess attention as recess duration increased *(F(2,24) = 13.08)*
US	27 children (age 50–63 months; 70% girls)	Recess duration of 10, 20 and 30 min		Secondary analyses:
4.5				Intervention effect was strongest following the 20 min recess and for girls
Van Cauwenberghe, 2012 [42]	Non-randomized trial	4 preschools	During recess	During recess
Belgium	128 children (age 4–6; 46% girls)	Decrease of playground density	I. min and % spent in sedentary time	I. Significant decrease after the intervention compared to pre-intervention *(χ*^*2*^*(2,N = 128) = 26.0, p < 0.001; χ*^*2*^*(2,N = 128) = 19.5, p < 0.001)*^*b*^
6.5			II. min and % spent in LMVPA	II. Significant increase after the intervention compared to pre-intervention *((χ*^*2*^*(2,N = 128) = 26.0, p < 0.001; χ*^*2*^*(2,N = 128) = 19.5, p < 0.001)*^*b*^
			III. min and % spent in MVPA	III. Significant increase after the intervention compared to pre-intervention *((χ*^*2*^*(2,N = 128) = 15.3, p < 0.001; χ*^*2*^*(2,N = 128) = 27.2, p < 0.001)*^*b*^
			During preschool time	During preschool time
			IV. min and % spent in sedentary time	IV. No significant difference after the intervention compared to pre-intervention
			V. min and % spent in LMVPA	V. No significant difference after the intervention compared to pre-intervention
			VI. min and % spent in MVPA	VI. No significant difference after the intervention compared to pre-intervention
			During the entire day	During the entire day
			VII. min and % spent in sedentary time	VII. No significant difference after the intervention compared to pre-intervention
			VIII. min and % spent in LMVPA	VIII. No significant difference after the intervention compared to pre-intervention
			IX. min and % spent in MVPA [accelerometer]	IX. Significant increase *(χ*^*2*^*(2,N = 107) = 5.8, p < 0.05)*^*b*^
				Secondary analyses:
				Intervention effect was stronger for girls compared to boys for the% spent in sedentary time and LMVPA
**PRIMARY SCHOOL INTERVENTIONS**				
Brink, 2010 [41]	Non-randomized controlled trial	9 primary schools	I. % active boys/girls on school grounds [SOPLAY]^2^	I. Significantly more active boys and girls in established and recently rebuilt schools compared to in control schools
US	5488 children (age 4–11; 48% girls)	Schoolyard renovations (installation of play equipment, asphalt areas for structured games, and a grassed multipurpose playfield) within the past year (3 schools=’recently rebuilt schools’)	II. % sedentary boys/girls on school grounds [SOPLAY]^2^	II. No significant differences in established and recently rebuilt schools compared to in control schools
8.5		Schoolyard renovations in place for at least 2 years (3 schools=’established schools’)	III. Energy expenditure rate (EER) on school grounds [calculated]	III. Significant higher EER in boys and girls in established and recently rebuilt schools compared to in control schools
		No renovations/minimal improvements over the years (3 schools=’control schools’)		Secondary analyses:
				Significantly more active boys when there was an unstructured hard surface
				Significant less sedentary behavior among girls in established and recently rebuilt schools compared to in control schools
				Significantly more active girls when there was a soft structured surface
Bundy, 2008 [43]	Non-randomized trial	1 primary school	Playfulness (score 0–3; 30 items) [ToP]^3^	Significant increase after the intervention *(ES = 0.55; 95% CI = −0.08,1.19)* compared to pre-intervention
Australia	20 children (age 5–7; 70% girls)	Introduction of play materials		
7.5				
Colabianchi, 2009 [40,46]	Non-randomized controlled trial	20 primary schools	I. % active children on school grounds	I. No significant differences in intervention schools compared to control schools
US	136 children	Renovation of playground (new play equipment, safety and site improvements) (10 schools)	II. % moderately active children on school grounds	II. No significant difference in intervention schools compared to control schools
8.5		No intervention (10 schools)	III. % vigorously active children on school grounds [SOPLAY]^2^	III. No significant difference in intervention schools compared to control schools
Huberty, 2011 [39]	Non-randomized trial	2 primary schools (public and parochial)	During recess	During recess
US	Public school:	Staff training, recreational equipment and playground markings (2 schools)	I. MPA (counts/min)	I. Significant increase after the intervention compared to pre-intervention
8.5	45 children (age 9.6; 42% girls)		II. VPA (counts/min)	II. Significant increase after the intervention compared to pre-intervention
	Parochial school:		During the school day	During the school day
	48 children (age 9.6; 50% girls)		III. MPA (counts/min)	III. Significant increase after the intervention compared to pre-intervention
			IV. VPA (counts/min) [accelerometer]	IV. Significant increase after the intervention compared to pre-intervention
Loucaides, 2009 [34]	RCT Randomization: school-level	3 primary schools (innercity)	I. Steps/min during recess	I. Significant increase in the intervention schools compared to the control school *(F(2,222) = 3.08)*
Cyprus	228 children (age 11.2; 50% girls)	Allocating play space for team games, playground markings and ropes for jumping (school 1)	II. Steps/min after school [pedometer]	II. No significant difference in the intervention schools compared to the control school
9		Allocating play space for team games (school 2)		
		No intervention (school 3)		
Ridgers, 2007 [28,29]	Non-randomized controlled trial 297 children (age 5–10; 50% girls)	26 primary schools	I. % time spent in MVPA during recess	I. Significant increase in intervention schools compared to control schools *(β = 5.95; 95% CI = 0.14,11.77)*
UK		Incentive for change of playground with use of playground markings and physical structures (15 schools)	II. % time spent in VPA during recess [accelerometer]	II. Significant increase in intervention schools compared to control schools *(β = 1.07; 95% CI = 0.01,3.39)*
8.5		No intervention (11 schools)		No significant effects when analyses were adjusted^c^
				Secondary analyses:
				Intervention effect was stronger for younger children and when recess duration increased
Ridgers, 2007 [28,29]	Non-randomized controlled trial	26 primary schools	I. % time spent in MVPA during recess	I. Significant increase in intervention schools compared to control schools^a^*(heart rate: β = 4.03; 95% CI = 0.15, 7.91), accelerometer: β = 4.53; 95% CI = 0.59, 8.47)*
UK	470 children (age 8.1-10.1; 51% girls)	Incentive for change of playground with use of playground markings and physical structures (15 schools)	II. % time spent in VPA during recess [heart rate telemetry, accelerometer]	II. Significant increase in intervention schools compared to control schools^a^*(heart rate: β = 2.34; 95% CI = 0.06, 4.80, accelerometer: β = 2.32; 95% CI = 0.71,3.93)*
7		No intervention (11 schools)		
Ridgers, 2010 [30,49]	Non-randomized controlled trial	26 primary schools	Morning recess	I-IV. No significant increase in intervention schools compared to control schools
UK	470 children (age 8.1-10.1; 51% girls)	Incentive for change of playground with use of playground	I. % time spent in MVPA	
8		markings and physical structures (15 schools)	II. % time spent in VPA	
		No intervention (11 schools)	Lunch recess	
			III. % time spent in MVPA	
			IV. % time spent in VPA [heart rate telemetry, accelerometer]	
Stratton, 2005 [32]	Non-randomized controlled trial	8 primary schools (4 early primary; 4 late primary)	I.% time spent in MVPA during recess	I. Significant increase in intervention schools compared to control schools *(F(1,204) = 13.7)*
UK	99 children (age 4–11; 49% girls)	Painting of playground markings (2 early primary and 2 late primary schools)	II. % time spent in VPA during recess [heart rate telemetry]	II. Significant increase in intervention schools compared to control schools *(F(1,204 = 4.05*
9		No intervention (2 early primary and 2 late primary schools)		^*c*^Secondary analyses:
				Increase in MVPA in late primary schools was more than double than that found in early primary schools
Stratton, 2000 [31]	Non-randomized controlled trial	2 early primary schools	I. % of playtime in MVPA	I-II. No significant differences in intervention schools compared to control schools
UK	47 children (age 5–7; 51% girls)	Playground markings and no play equipment allowed on playground (except for a single football) (1 school)	II. % of playtime in VPA [heart rate telemetry]	
8.5		No playground markings and limited play equipment allowed (1 school)		
Verstraete, 2006 [35]	RCT	7 primary schools	Morning recess	Morning recess
Belgium	Randomization: school-level	Presentation and provision of game equipment (two jump ropes, two double Dutch ropes, two scoop sets, two	I. % time spent in LPA	I. No significant difference in intervention schools compared to control schools
9	235 children (age ±10.8; 49% girls)	scoop sets, two flying discs, two catch balls, one poco ball, one	II. % time spent in MPA	II. Significantly higher in intervention schools compared to control schools *(F(4) = 10.6)*^*d*^
		plastic ball, two plastic hoops, two super grips, three juggling	III. % time spent in VPA	III. No significant difference in intervention schools compared to control schools
		scarves, six juggling rings, six juggling bean balls, one diabolo,	IV. % time spent in MVPA	IV. No significant difference in intervention schools compared to control schools
		one angel-stick, four spinning plates, two sets of badminton	Lunch break	Lunch break
		racquets and two sets of oversized beach paddles) and activity cards with examples of games and activities that can be performed with the equipment (4 schools)	V. % time spent in LPA	V. No significant difference in intervention schools compared to control schools
			VI. % time spent in MPA	VI. Significantly higher in intervention schools compared to control schools *(F(4) = 28.3)*^*d*^
		No intervention (3 schools)	VII. % time spent in VPA	VII. Significantly higher in intervention schools compared to control schools (*F(4) = 13.1)*^*d*^
			VIII. % time spent in MVPA [accelerometer]	VIII. Significantly higher in intervention schools compared to control schools *(F(4) = 44.2)*^*d*^
				Secondary analyses:
				Girls spent significantly more time in LPA *F(4) = 2.4)*^*d*^, MPA *(F(4) = 2.2)*^*d*^*,* VPA (*F(4) = 0.5)*^*d*^ and MVPA *(F(4) = 2.9)*^*d*^ during morning recess

PA = physical activity; LPA = light intensity physical activity; MPA = moderate intensity physical activity; VPA = vigorous intensity physical activity; MVPA = moderate-to-vigorous physical activity; Methodological quality was assessed on a scale 0–14; ^1^OSRAP = Observational System for Recording Physical Activity in Preschoolers; ^2^SOPLAY = System for Observing Play and Leisure Activity in Youth; ^3^ToP = Test of Playfulness, an observational assessment of playfulness. Analyses were adjusted for ^a^age, gender, baseline physical activity levels and recess time; ^b^play duration and body mass index; ^c^sex, age, body mass index and recess duration; ^d^sex, age and accelerometer wear time; ^e^gender, day of accelerometry; ^f^gender and baseline MVPA. If no superscript number, the results of analyses were unadjusted.

**Table 2:** Characteristics of observational studies (n = 17) and associations of (pre)school playgrounds with children’s health

**Study;**	**School(s) type**	**Playground variables (unit) measurement**	**Outcome (unit)**	**Associations**^**1**^
**Country;**	**Study population (mean age/range; % girls**			
**Methodological quality**				
**PRESCHOOLS**				
Boldemann, 2006 [58]	11 preschools	Play potential/outdoor play environment score (sum of scores, divided by 3 and dichotomized into high if >2 and low if <2):	PA during school time (steps/min) [pedometer]	Significant increase of step count by 3.6 steps/min (p < .001)^a^
Sweden	197 children (age 4–6; 43% girls)	Total outdoor area (small if <2000 m^2^, medium if 2000–6000 m^2^, large if >6000 m^2^) [Department of Infrastructure, Stockholm Royal Institute of Technology, survey]		
9		Overgrown surface (trees, shrubbery) and broken ground (little/nonexistent, <half of area, >half of area) [observation]		
		Integration of play structures with vegetation		
Brown, 2009 [37,47]	24 preschools	Outdoor activity contexts [OSRAC-P]^1^	Sedentary activity, MVPA on school playground (% of intervals in which sedentary activity, MVPA is performed) [OSRAC-P]^1^	Compared to sociodramatic props, a child is significantly more likely to engage in MVPA if playing with balls/objects *(OR = 3.21; 95% CI = 2.54-4.05)*^*b*^, playing in open space *(OR = 2.57; 95% CI = 2.08-3.16)*^*b*^, and playing with fixed equipment *(OR = 1.31; 95% CI = 1.06-1.62)*^*b*^
US	476 children (age 3–5; 49% girls)	Presence of balls/objects		Compared to sociodramatic props, a child is significantly more likely to engage in sedentary activity if playing with balls/objects *(OR = 2.51; 95% CI = 2.15-2.91)*^*b*^, playing in open space *(OR = 2.29; 95% CI = 2.02-2.59)*^*b*^, and playing with fixed equipment *(OR = 2.41; 95% CI = 2.03-2.87)*^*b*^
8		Presence of open space		
		Fixed equipment		
		Presence of sociodramatic props		
		Presence of wheel toys		
Cardon, 2008 [57]	39 preschools	Playground features [observation]:	PA levels during recess (step counts/min)	Girls:
Belgium	783 children (age 5.2; 47% girls)	Children/m^2^	[pedometer]	Significant association of number of children/m^2^*(β =-5.411; SE=2.163),* number of supervising teachers *(β =-0.526; SE=0.239)*, and recess duration *(β =-0.001; SE=0.000)* with PA levels during recess
7.5		Supervision (number of teachers)		Boys:
		Aiming equipment (count)		Significant association of number of children/m^2^*(β = −4.635; SE = 2.104)*, recess duration *(β = −0.001; SE = 0.000)* with PA levels during recess
		Playing equipment (count)		
		Recess duration		
		Soft surface (0–1)		
		Markings		
		Vegetation		
		Height differences		
		Toys availability (0–1)		
Dowda, 2009 [45]	20 preschools	Fixed and portable equipment (count) [observation]	Sedentary activity on week and weekend days (min/h) [accelerometer]	Significant fewer sedentary time *(p = 0.05)*^*c*^ and more time spent in MVPA *(p = 0.03)*^*c*^ in schools wherein PA is promoted if more than 1 piece of portable equipment available
US	299 children (age 3-5; 50% girls)	Playground size (feet^2^) [measured]	Time spent in MVPA (min/h) [accelerometer]	Significant fewer sedentary time *(p < 0.01)*^*c*^ and more time spent in MVPA *(p = 0.02)*^*c*^ in schools wherein PA is promoted if less fixed playground
5				Significant fewer sedentary time *(p = 0.02)*^*c*^ and more time spent in MVPA *(p = 0.02)*^*c*^ in schools wherein PA is promoted if larger playgrounds
Gubbels, 2012 [60]	9 preschools	Portable and fixed equipment (count) [EPAO]^2^:	Outdoor PA level during school time (1–5) [OSRAC-P]^1^	Significant association of portable jumping equipment *(β = 0.36),* portable slides *(β = −0.55)*, fixed structured track *(β = 0.53),* fixed sandbox *(β = −0.49),* fixed swinging equipment *(β = −0.41),* and age *(β = 0.13)* with outdoor PA levels
The Netherlands	175 children (age 2.6; 49% girls)	*Portable*: balls, climbing structures, floor play equipment, jumping equipment, push/pull toys, riding toys, slides, sand/water toys, twirling equipment		Significant association of fixed structured track with outdoor PA levels *(β* ***=*** *0.23)*^*d*^
8.5		*Fixed*: structured track, merry-go-round, climbing structures, see-saw, slides, tunnels, balancing surfaces, sandbox, swinging equipment		
**PRIMARY, SECONDARY, MIXED SCHOOLS**				
Colabianchi, 2011 [40,46]	20 primary schools	Playground features on renovated schoolyards [EAPRS]^3^:	PA levels on school grounds (% active, vigorously active and moderately active) [SOPLAY]^4^	No significant association of any of the play features with PA levels on school grounds^e^
US	185 children (47% girls)	Total unique types of play equipment (0–10)		
9		Number of play features (0-∞)		
		Overall condition (1–3)		
		Overall cleanliness (1–3)		
		Overall quality (0–1)		
		Overall safety (0–1)		
		Presence of benches (0–1)		
		Presence of trash cans (0–1)		
		Coverage/shade for resting features (0–3)		
		Renovated (0–1)		
Fairclough, 2012 [50]	8 primary schools	Playground area (m^2^/student)	Daily PA levels (count/min, min spent in MPA, min spent in VPA)	Significant positive association of playground area with MPA before school *(β = 0.15; SE = 0.06),* lunchtime MPA *(β = 0.5; SE = 0.2),* and school time MPA *(β = 0.8; SE = 0.3)*
UK	223 children (age 10.7; 56% girls)		PA levels at school time, out of school, before school, during class time, during recess, during lunchtime, after school (min spent in MPA, min spent in VPA) [accelerometer]	Boys:
9				Engagement of greater MPA during recess than girls *(β = 1.4; SE = 0.5)*
McKenzie, 2010 [48]	13 primary schools	Potential areas for PA with:	PA during play and leisure (% sedentary, walking, vigorous and MVPA) [SOPLAY]^4^	Significant association of no supervision with walking *(boys: OR = 0.49; 95% CI = 0.36,0.66, girls: OR = 0.25; 95% CI = 0.15,0.41*) and engaging in MVPA *(boys: OR = 0.31; 95% CI = 0.21,0.47, girls: OR = 0.56; 95% CI = 0.38,0.82)* compared to supervised areas
US	36,955 children (54% girls)	Supervision (0–1)		Significant association of areas with play equipment and engaging in MVPA *(boys: OR = 9.27; 95% CI = 6.07,14.15, girls: OR = 2.94; 95% CI = 2.04,4.24)*
7.5		Available equipment (0–1)		Boys:
		Organized activities (0–1)		Engaged in greater MVPA compared to girls in unsupervised areas *(boys: OR = 0.31; 95% CI = 0.21,0.47, girls: OR = 0.56; 95% CI = 0.38,0.82)*
		Time period (before school, recess, lunch)		Engaged in greater MVPA compared to girls in areas with play equipment (see main results above)
				Girls:
				Engaged in less MVPA compared to boys in areas with organized activities *(boys: OR = 0.59; 95% CI = 0.41,0.85, girls: OR = 0.54; 95% CI = 0.37,0.81)*
Nielsen, 2010 [55]	7 primary schools	Playground surface area (m^2^) [measuring tape]	PA levels at home, during school time and total (% time spent in MPA, VPA, MVPA, average counts/min) [accelerometer]	Significant association of number of play facilities and both total PA and school time PA in average counts/min *(OR = 1.038; 95% CI = 1.025,1.051) (OR = 1.027; 95% CI = 1.012,1.041)*^*f*^
New Zealand	417 children (age 5–12; 48% girls)	Number of permanent play facilities [self-report]		Significant association of number of play facilities and school time spent in VPA *(OR = 1.101; 95% CI = 1.072,1.132)*^*f*^
9				Significant association of number of play facilities and total time spent in both MVPA and VPA *(OR = 1.102; 95% CI = 1.066,1.139) and (OR = 1.034; 95% CI = 1.015,1.054)*^*f*^
Nielsen, 2012 [59]	18 pre/primary schools	Permanent play facilities (number)	School time and total PA (average counts/min, min/day in MPA or MVPA, % active < 1 hour/day,% vigorously active <1.5 hours/day) [accelerometer]	Preschools:
Denmark	Time point 1:	Playground area (m^2^)		Significant association of an increase of permanent play facilities with school time PA *(average counts: OR = 1.0139; 95% CI = 1.0093,1.0186, time in MPA: OR = 1.0257; 95% CI = 1.0186,1.0328, time in MVPA: OR = 1.0257; 95% CI = 1.0186,1.0351)*^*g*^
7.5	594 children (age 6–7; 48% girls)			Significant association of an increase of play facilities with total PA *(average counts: OR = 1.0069; 95% CI = 1.0043,1.0106, time in MPA: OR = 1.0067; 95% CI = 1.0023,1.0116, time in MVPA: OR = 1.0077; 95% CI = 1.0046,1.0116)*^*g*^
	Time point 2:			Primary schools:
	518 children (age 9–10; 49% girls)			Significant association of an increase of permanent play facilities with school time PA *(average counts: OR = 1.0261; 95% CI = 1.0199,1.0324, time in MPA: OR = 1.0194; 95% CI = 1.0124,1.0257, time in VPA: OR = 1.0373; 95% CI = 1.0239,10.513, time in MVPA: OR = 1.0238; 95% CI = 1.0131,1.0295)*^g^
				Significant association of an increase of play facilities with total PA (*average counts: OR = 1.0094; 95% CI = 1.0054,1.0134, time in MPA: OR = 1.0093; 95% CI = 1.0035,1.0139, time in MVPA: OR = 1.0093; 95% CI = 1.0041,1.0133)*^*g*^
Ridgers, 2010 [30,49]	8 primary schools	Playground characteristics [Google Earth Pro software]:	PA levels during recess (% time spent in sedentary, moderate and vigorous activity levels) [SOCARP]^5^	Significant association of equipment provision with sedentary activity
UK	128 children (age 9–10; 61% girls)	Playground size (m^2^)		*(β = −8.15; 95% CI = −16.28,-0.02)*^*h*^ and moderate activity *(β = 6.91; 95% CI = 0.21,13.61)*
6.5		Play space (number of children per m^2^ during recess)		Significant association of play space with sedentary activity *(β = −2.70; 95% CI = −3.88,-1.52)*^*i*^ and vigorous activity *(β = 2.02; 95% CI = 1.20,2.84)*^*j*^
		Fixed equipment (count)		Girls:
		Playground markings (count)		Engaged in greater sedentary activity and less vigorous activity *(β = 13.83; 95% CI = 7.14,20.5)*^*k*^*(β = −8.22; 95% CI = −12.49,-3.95)*^*l*^
		Seating (count)		
		Supervision (number of adults)		
		Recess duration (min)		
Taylor, 2011 [56]	21 primary schools	Number of permanent play facilities (playground count: 30–135) [observations]	PA in recess, at school, at home and total (average counts/min, min of MVPA/day) [accelerometer]	Significant association of number of playground facilities and PA during recess *(average counts: β = 3.2; 95% CI = 0.0,6.4, MVPA: β = 8.3; 95% CI = 0.8,16.3)*^*m*^
New Zealand	441 children (age 8; 47% girls)			Significant association of number of playground facilities and PA at home *(average counts: β = 5.6; 95% CI = 3.5,7.7, MVPA: β = 10.5; 95% CI = 5.5,15.7)*^*m*^
7.5				No significant association of number of playground facilities and PA at school
Willenberg, 2010 [51]	23 primary schools (governmental, independent, religious and special development)	Playground characteristics [observation]:	PA before school, in recess and after school on school playground (% time spent in sedentary, MPA and VPA) [SOPLAY]^4^	Significant association of loose equipment and teacher supervision with time spent in VPA
Australia	3006 children (50% girls)	Loose equipment (0–1)		Significant association of fixed play equipment, court markings/goals and play markings with time spent in MPA
5.5		Supervision (0–1)		
		Surface type (grass-bitumen)		
		Fields (no improvements-with boundary lines/goals)		
		Fixed play equipment (0–1)		
		Bitumen (no improvements-with boundary lines/goals-with play markings)		
Zask, 2001 [52]	18 primary schools	Playground characteristics during recess and lunch [CAST]^6^:	PA levels in school break times (% engaged in MVPA and VPA) [CAST]^6^	Significant association of school size and MVPA and VPA levels *(MVPA: coefficient = −0.121; SE = 0.053, VPA: coefficient = −0.164; SE = 0.063)*
Australia	3912 children (age maximum 6)	Equipment availability/use		Significantly lower MPVA and VPA levels during recess than during lunch periods *(MVPA: coefficient = −0.149; SE = 0.076, VPA: coefficient = −0.296; SE = 0.097)*
9.5		Teacher presence/behavior		Significant (one-tailed) association of balls-to-child ratio and VPA levels *(coefficient = 0.019; SE = 0.010)*
				Girls:
				Engaged in less MVPA and VPA than boys *(MVPA: coefficient = −0.413;SE = 0.053, VPA: coefficient = −0.552; SE = 0.081)*
Haug, 2008 [54]	68 secondary schools	Playground facilities:	Participation in recess PA (1–5) [self-report]	Significant association of playground facilities with recess PA *(OR = 4.49; 95%CI = 1.93,10.44)*^*n*^
Norway	1347 children (age 13; 48% girls)	Environmental index (comprised a set of 16 natural or built characteristics of indoor school area, schoolyard or school neighborhood)		Significant association of open fields *(OR = 4.31; 95% CI = 1.65,11.28),* outdoor obstacle course *(OR = 1.78; 95% CI = 1.32,2.40)*, and playground equipment *(OR = 1.73; 95% CI = 1.24,2.42)* with recess PA
8				
Haug, 2010 [53]		Characteristics of school environment (present yes/no) [self-report]:	PA level during recess [self-report]	In secondary schools:
Norway	130 schools (80 primary; 21 secondary; 29 combined)	Soccer field		Significant association of larger number of outdoor facilities with PA levels for boys and girls at secondary level compared to children in schools with fewer facilities *(OR = 2.69; 95% CI = 1.21,5.98 and OR = 2.90; 95% CI = 1.32,6.37)*
8.5	16,471 children (age 8–15)	Areas for other ball games		Boys:
		Areas for hopscotch/skipping rope		Significant association of areas for hopscotch/skipping rope *(OR = 2.53; 95% CI = 1.55,4.13),* with a soccer field *(OR = 1.68; 95% CI = 1.15,2.45),* with playground equipment *(OR = 1.66; 95% CI = 1.16,2.37),* and with a sledding hill *(OR = 1.70; 95% CI = 1.23,2.35)* with higher PA levels compared to children in schools with fewer facilities
		Playground equipment		Girls:
		Outdoor obstacle course		Significant association of a sledding hill with PA levels *(OR = 1.58; 95% CI = 1.11,2.24)*
		Sledding hill		No significant associations were found in primary schools.
		Green spaces/forest areas		
		Areas for boarding skating		
		Outdoor facility index (0–1)		
Sallis, 2001 [44]	24 middle schools	Characteristics of activity areas [observation]:	MVPA (% spent in MVPA) before school, during school, during lunch, after school [SOPLAY]^4^	Girls:
US	25,944 children	Area type (courts space with permanent markings, open field space with no markings, indoor activity space including multipurpose rooms and gymnasiums)		Significant more time spent in MVPA when equipment was available (F = 4.68)^o^
5.5		Area size (m^2^) [measurement]		Significant more time spent in MVPA when school environments had high levels of improvements and supervision (F = 15.15)^o^42% of the variance in MVPA explained by environmental variables
		Permanent improvements (number of basketball hoops, tennis courts, baseball diamonds and football/soccer goals)		Boys:
		Equipment (0–1)		Significant more time spent in MVPA when supervision was present (F = 3.11)^o^ and if equipment was available (F = 11.91)^o^
		Supervision (0–1)		Significant more time spent in MVPA when areas had high levels of both improvements and supervision (F = 12.01)^o^
				59% of the variance in MVPA explained by environmental variables

PA = physical activity; LPA = light intensity physical activity; MPA = moderate intensity physical activity; VPA = vigorous intensity physical activity; MVPA = moderate-to-vigorous physical activity; Methodological quality was assessed on a scale 0–11; IRR = Incident Rate Ratio; 95%CI = 95% confidence interval; ^1^OSRAC-P = Observational System for Recording Physical Activity in Children-Preschool Version; ^2^EPAO = Environment and Policy Assessment and Observation Instrument; ^3^EAPRS = Environmental Assessment of Public Recreation Spaces; ^4^SOPLAY = System for Observing Play and Leisure Activity in Youth; ^5^SOCARP = System for Observing Children’s Activity and Relationships during Play; ^6^CAST = Child Activity Scanning Tool. Analyses adjusted for: ^a^gender; ^b^gender, age, ethnicity, BMI; ^c^BMI, race, gender, age, parental education, preschool; ^d^portable jumping equipment, portable slides, fixed sandbox, fixed swinging equipment, and age; ^e^overall safety, presence of benches, and coverage/shade for resting features; ^f^age, gender, staffing and school roll, PA policies and weather; ^g^season, gender and socio-economic status; ^h^gender and play space; ^i^gender and equipment; ^j^gender and temperature; ^k^play space and equipment; ^l^ temperature and play space; ^m^ age, gender and school roll; ^n^socio-economic status, gender and interests in school PA; ^o^characteristics of activity areas (independent variables), percentage of non-White students, percentage receiving subsidized lunch, percentage bused, school start time, school end time, and mean parental education. If analyses were unadjusted, no superscript number is added.

#### Experimental studies

The 16 publications reporting on experimental studies were based on 13 studies, since some publications were based on the same study sample. This was true for three publications of Ridgers and colleagues, in which the effects of incentives to change playgrounds on physical activity during recess were reported for different follow-up periods, i.e., six weeks, six months, and 12 months [28-30]. Further, Stratton and colleagues reported twice on the effects of painting markings on playgrounds of two early primary schools; once in 2000 and once again in a more recent publication in 2005. In the most recent publication, the sample size had increased through the inclusion of two additional late primary schools [31,32].

Seven of the 13 experimental studies (54%) included a control group in the study design, and only three of them used a randomization procedure to allocate schools and/or children to an intervention or control condition [33-35]. Six of the 13 experimental studies were conducted in the United States [36-41]. The remaining seven studies were conducted in Belgium [33,35,42], United Kingdom [28-32], Cyprus [34], and Australia [43]. All study samples contained approximately 50 percent girls. Sample sizes ranged from one to 40 schools and from five to 5488 children. Five studies specifically targeted preschools with children from three to six years old [33,36-38,42], whereas eight studies described the effects of playground interventions on primary schools, with children aged four to 11 years old [28-32,34,35,39-41,43].

In preschools, the five interventions included the provision of play equipment [33,36], the promotion of physical activity on playgrounds by teachers [37], variations in recess duration [38], and variations in playground density (m^2^/child) [42]. Two of the five experimental studies included a control group, i.e., Cardon et al. [33] and Brown et al. [37]. Cardon and colleagues [33] compared the effects of two intervention conditions (i.e., the provision of play equipment only and the provision of play equipment and painting of playground markings) with a no-intervention condition. Brown et al. [37] also tested two intervention conditions (i.e., the promotion of physical activity by teachers against the addition of group discussions and the provision of stickers when children showed sufficient physical activity) against a no-intervention condition.

In primary schools, all eight interventions included the provision of play equipment and/or the application of playground markings. Six of the eight experimental studies in primary schools included a no-intervention condition [28-32,34,35,40,44]. One study examined the isolated effect of playground markings with a no-intervention condition [31,32]. Similarly, Bundy et al. [43] evaluated the effect of the provision of play materials, only without a control condition. In the other studies the interventions contained multiple components. Of these six studies, Hyberty et al. [39] was the only study without a control condition. Interventions in two studies contained both the provision of play equipment and the application of playground markings, complemented with the creation of space for team games, and staff training respectively [34,39]. Ridgers et al. [28-30], Colabianchi et al. [40], and Brink et al. [41] combined the provision of play equipment and playground markings with playground improvements. Verstraete and colleagues provided both play equipment and activity cards which informed children on the activities that could be performed with different pieces of play equipment [35].

As is shown in Table 1, nearly all experimental studies (n = 11) evaluated intervention effects on time spent in different levels of physical activity during recess. The proportion of time spent in light, moderate and vigorous physical activity and sedentary activity was mostly assessed with the use of accelerometers, with the exception of three studies in which physical activity was assessed by observations [37,40,41], one study that used pedometers [34], and one study that used heart rate telemetry [31,32]. Other observed outcomes were playfulness [43] and post-recess attention [38]. Intervention effects were assessed mostly directly during recess. In case of the provision of playground markings and structural playground improvements, outcomes were assessed with follow-up periods ranging from one month to 12 months.

#### Observational studies

Of the 33 included publications, 17 reported on observational studies. Their study characteristics are summarized in Table 2. Five of the 17 observational studies were conducted in the United States [44-48] and the remaining studies were conducted in the United Kingdom [49,50], Australia [51,52], Norway [53,54], New Zealand [55,56], Belgium [57], Sweden [58], Denmark [59], and the Netherlands [60]. Forty-three to 61 percent of the study samples were girls, and sample sizes ranged from seven to 130 schools and from 128 to 36.955 children. Five studies specifically reported on associations in preschools (age range: 2.6 to six years old), nine studies in primary schools (age range: five to 12 years old), and three in other types of schools, such as in secondary schools, middle schools or mixed schools (age range: eight to 15 years old). The study of Nielsen et al. [59] was exceptive, as in this study the association of playground characteristics with physical activity was examined at two time points: at preschool, and at primary school.

Many different playground characteristics were examined and categorized as either hardware, software, or orgware. With regard to software, some studies made a distinction between fixed and loose equipment [45,49,51,60]. Playground characteristics were assessed through self-reports [53,55] or observations [44-52,54,56-60], mostly with the use of validated instruments, such as the Environmental Assessment of Public Recreation Spaces Tool (EAPRS), Children Activity Scanning Tool (CAST), and Environment and Policy Assessment and Observation instrument (EPAO) [46,52,60]. In accordance with the experimental studies, the outcomes reported in observational studies were mainly in terms of time spent in different levels of physical activity during recess. Physical activity was mostly assessed through observational instruments [44,46-49,51,52,60], four studies used an accelerometer [45,50,56,59], three studies used a self-report instrument [53-55], and two studies used a pedometer [57,58]. In addition to physical activity, Colabianchi et al. [46] also assessed the utilization of the playground through observations.

#### Methodological quality

The methodological quality scores of the experimental studies ranged from 4.5 to 10 points on a 14-point scale. Two of the 13 studies (15%) demonstrated high methodological quality [33,41], eight studies (62%) were of moderate quality [28-32,34-36,39,40,43], and three studies (23%) were of low quality [37,38,42]. None of the experimental studies reported on the use of blinding of study participants, personnel who implemented the intervention or outcome assessors. A summary of the methodological quality analysis per study can be found in Additional file 2. The methodological quality of the observational studies ranged from 5.0 to 9.5 points on an 11-point scale. Seven of the 17 observational studies (41%) demonstrated high methodological quality [46,50,52,53,55,58,60], nine studies (53%) were of moderate quality [30,37,44,48,51,54,56,57],[59], and one study (6%) was of low quality [45]. Information on the reliability and validity of instruments for the measurement of playground characteristics was reported by only one and five studies respectively. Detailed information on the quality scores of the observational studies can be found in Additional file 2.

### Study outcomes

#### Experimental studies

Although it was our intention to summarize the effects of (pre)school playgrounds on children’s health in terms of physical activity, cognitive, and social outcomes, nearly all experimental studies (n = 11) focused on physical activity as the primary outcome. Only one study examined the effect a playground-based intervention on cognitive outcomes (i.e., post-recess attention, Holmes et al. [38]) and social outcomes (i.e., playfulness, Bundy et al. [43]).

In preschools, the effect of decreased playground density was investigated by one study that showed a significant increase of physical activity levels (see Tables 1 and 3) [42]. The provision of play equipment showed mixed effects on physical activity levels of preschool children [33,36]. No effects were found in the two studies that investigated the provision of playground markings and promotion of physical activity by teachers [33,37]. Increase of recess duration was investigated by one single study that showed positive effects on post-recess attention of preschool children [38]. Further, two studies investigated the effects of a multi-component intervention, including a combination of promotion of physical activity by teachers and guided discussions in one study, and the provision of play equipment and playground markings in another study [33,37]. Both studies showed no beneficial effects on preschool children. In summary, taking into account the study design, the sample size and the methodological quality of the experimental studies, there is inconclusive evidence for an effect of the provision of play equipment, playground markings or for multi-component interventions at preschools on children’s health (Table 3). No evidence was generated for the other intervention strategies at preschools.

**Table 3:** **Summary of level of evidence for the intervention strategies used in the included experimental studies**^**a**^

**PRESCHOOL**					**PRIMARY SCHOOL**				**Total**
**Intervention strategy**	**Studies that investigated the intervention strategy**	**Number of studies**	**Studies that found significant positive effect [n(%)]**	**Level of evidence**	**Studies that investigated the intervention strategy**	**Number of studies**	**Studies that found significant positive effect [n(%)]**	**Level of evidence**	**Level of evidence**
Decreased playground density	Van Cauwenberghe, 2012 [42]	1	1 (100)	No evidence		0	0 (10)	No evidence	No evidence
Provision of play equipment	Hannon, 2008 [36]	2	1 (100)	Inconclusive	Bundy, 2008 [43]	2	2 (100)	Inconclusive	Moderate
	Cardon, 2009 [33]				Verstraete, 2006 [35]				
Playground markings	Cardon, 2009 [33]	1	1 (100)	Inconclusive	Stratton, 2000 [31]	2	1 (50)	Inconclusive	Inconclusive
					Stratton, 2005 [32]				
Promotion by staff	Brown, 2009 [37,47]	1	0 (10)	No evidence		0	0 (10)	No evidence	No evidence
Increase of recess duration	Holmes, 2006 [38]	1	1 (100)	No evidence		0	0 (10)	No evidence	No evidence
Allocating play space for team games		0	0 (10)	No evidence	Loucaides, 2009 [34]	1	1 (100)	Inconclusive	Inconclusive
Multicomponent	Brown, 2009 [37,47]	2	0 (10)	Inconclusive	Huberty, 2011 [39]	7	4 (57)	Moderate	Inconclusive
	Cardon, 2009 [33]				Loucaides, 2009 [34]				
					Ridgers, 2007 [28,29]				
					Ridgers, 2007 [28,29] (Longterm)				
					Ridgers, 2010 [30,49]				
					Brink, 2010 [41]				
					Colanbianchi, 2009 [40,46]				

^a^Level of evidence was based on study design, study quality and sample size according to a flow chart of decision-making for level of evidence [16].

In primary schools, the two interventions that targeted the provision of play equipment were effective with regard to physical activity levels during recess and playfulness (see Tables 1 and 3) [35,43]. The two studies that investigated the effects of the provision of playground markings found mixed effects [31,32]. Allocating play space for team games was investigated by one study that found a significant beneficial effect [34]. Seven studies investigated multi-component playground interventions, of which the majority showed beneficial effects on physical activity levels [29,34,39,41]. Multi-component interventions that showed beneficial effects contained combinations of staff training, play equipment and playground markings [39], the allocation of play space, play equipment and playground markings [34], the provision of playground markings and physical structures [28-30] and the installation of play equipment and asphalt areas [41] respectively. When summarizing the effects into levels of evidence, one can conclude from Table 3 that there is inconclusive evidence for an effect of the provision of play equipment, playground markings and allocating play space in primary schools on children’s physical activity level. Moderate evidence is found for an effect of the use of multi-component intervention strategies. For the remaining separate intervention strategies no evidence is found.

When the evidence for different playground-based intervention strategies is summarized regardless of school type (preschool or primary school), studies generate moderate evidence for an effect of the provision of play equipment, inconclusive evidence for an effect of the use of playground markings, allocating play space or multi-component interventions, and no evidence for an effect of decreasing playground density, the promotion of physical activity by staff and increasing recess duration on children’s health in terms of physical activity, cognitive, and social outcomes.

#### Subgroups

With regard to subgroups in which playground interventions are most effective, four experimental studies found stronger effects on the physical activity level and on post-recess attention of girls compared to boys. These interventions entailed the decrease of playground density [42], the presentation and provision of game equipment and activity cards [35], and variations in recess duration [38]. Brink et al. [41] indicated that girls were more active on soft structured surfaces (with play equipment, fall zones and play fields with grass), in contrast to boys who were more active on hard unstructured surfaces (unprogrammed creative play, educational marking areas, sitting and social gathering areas, shade areas). Subgroup analyses in observational studies indicated a more pronounced association between less supervising teachers and physical activity levels in girls [57]. In boys, associations of physical activity levels with the presence of hopscotch and skipping rope areas [53], decreased playground density [57], and the presence of soccer fields [53] were more prominent.

#### Observational studies

In Table 4 the associations with physical activity are summarized per type of playground characteristic: hardware, software, and orgware. In preschools, summary codes of association indicate that there is evidence for a positive association between physical activity and playground size (++), the presence of an open field with no markings (++), a structured track (+++), decreased playground density (++), and increased recess duration (++). Negative associations were found for the presence of slides (- - -), a sandbox (- - -), swinging equipment (- - -), and supervision (−−).

**Table 4:** Summary of associations between playground characteristics and physical activity in (pre)school children, according to included observational studies

	**Preschool**				**Primary, secondary, mixed school**				**Total**
	**Studies that investigated the playground characteristic**	**Number of studies**	**Studies that found significant positive or negative associations (n (%))**	**Summary code of association**	**Studies that investigated the playground characteristic**	**Number of studies**	**Studies that found significant positive or negative associations (n (%))**	**Summary code of association**	**Summary code of association**
**HARDWARE**									
Playground size	Boldemann, 2006 [58]	2	2 (100)	++	Fairclough, 2012 [50]	5	1 (20)	0	?
	Dowda, 2009 [45]				Nielsen, 2010 [55]				
					Nielsen, 2012 [59]				
					Ridgers, 2010 [30,49]				
					Sallis, 2001 [44]				
Surface type	Cardon, 2008 [57]	1	0 (0)	0	Willenberg, 2010 [51]	1	0 (0)	0	0
Surface with vegetation/green	Boldemann, 2006 [58]	2	1 (50)	?	Haug, 2008 [54]	2	0 (0)	0	0
	Cardon, 2008 [57]				Haug, 2010 [53]				
**SOFTWARE**									
Coverage/shade		0	0 (0)	0	Colabianchi, 2011 [40,46]	1	0 (0)	0	0
Play equipment (unspecified)		0	0 (0)	0	McKenzie, 2010 [48]	7	6 (86)	++	++
					McKenzie, 2010^♂^[48]				
					Haug, 2008 [54]				
					Haug, 2010^♂^[53]				
					Haug, 2010 [53]				
					Sallis, 2001 [44]				
					Colabianchi, 2011 [40,46]				
Lack of cleanliness		0	0 (0)	0	Colabianchi, 2011 [40,46]	1	0 (0)	0	0
Safety		0	0 (0)	0	Colabianchi, 2011 [40,46]	1	0 (0)	0	0
Quality		0	0 (0)	0	Colabianchi, 2011 [40,46]	1	0 (0)	0	0
Balls	Brown, 2009 [37,47]	2	1 (50)	?	Zask, 2001 [52]	1	1 (100)	+++	+++
	Gubbels, 2012 [60]								
Climbing structures	Gubbels, 2012 [60]	1	0 (0)	0		0	0 (0)	0	0
Floor play equipment	Gubbels, 2012 [60]	1	0 (0)	0		0	0 (0)	0	0
Jumping equipment	Gubbels, 2012 [60]	1	0 (0)	0		0	0 (0)	0	0
Portable equipment (unspecified)	Dowda, 2009 [45]	2	1 (50)	?	Willenberg, 2010 [51]	1	1 (100)	++	++
	Cardon, 2008 [57]								
Push/pull toys	Gubbels, 2012 [60]	1	0 (0)	0		0	0 (0)	0	0
Riding toys	Gubbels, 2012 [60]	2	0 (0)	0		0	0 (0)	0	0
	Brown, 2009 [37,47]								
Sand/water toys	Gubbels, 2012 [60]	1	0 (0)	0		0	0 (0)	0	0
Slides	Gubbels, 2012 [60]	1	1 (100)	- - -		0	0 (0)	0	- - -
Sociodramatic props	Brown, 2009 [37;47]	1	0 (0)	0		0	0 (0)	0	0
Twirling equipment	Gubbels, 2012 [60]	1	0 (0)	0		0	0 (0)	0	0
Balancing surfaces	Gubbels, 2012 [60]	1	0 (0)	0		0	0 (0)	0	0
Benches and seating		0	0 (0)	0	Ridgers, 2010 [30,49]	2	0 (0)	0	0
					Colabianchi, 2011 [40,46]				
Climbing structures	Gubbels, 2012 [60]	1	0 (0)	0	Haug, 2008 [54]	1	0 (0)	0	0
Fixed equipment (unspecified)	Brown, 2009 [37,47]	2	1 (50)	?	Taylor, 2011 [56]	7	6 (86)	+++	+++
Permanent play facilities/improvements	Dowda, 2009 [45]				Willenberg, 2010 [51]				
					Nielsen, 2010 [55]				
					Nielsen, 2012 [59]				
					Ridgers, 2010 [30,49]				
					Sallis, 2001 [44]				
					Colabianchi, 2011[40,46]				
Hopscotch/skipping rope area		0	0 (0)	0	Haug, 2010 ^♂^[53]	1	1 (100)	+++	+++
Merry-go-round	Gubbels, 2012 [60]	1	0 (0)	0		0	0 (0)	0	0
Obstacle course		0	0 (0)	0	Haug, 2008 [54]	2	1 (50)	?	?
					Haug, 2010 [53]				
Open field with no markings	Brown, 2009 [37,47]	1	1 (100)	++	Haug, 2008 [54]	2	1 (50)	?	++
					Sallis, 2001 [44]				
Room for cardio/weightlifting		0	0 (0)	0	Haug, 2008 [54]	2	0 (0)	0	0
Gym/sports hall					Sallis, 2001 [44]				
Sandbox	Gubbels, 2012 [60]	1	1 (100)	- - -		0	0 (0)	0	- - -
See-saw	Gubbels, 2012 [60]	1	0 (0)	0		0	0 (0)	0	0
Ski/skateboard/skating facilities		0	0 (0)	0	Haug, 2010 [53]	3	1 (33)	0	0
Sledding hill					Haug, 2008 [54]				
					Haug, 2010 [53]				
Slides	Gubbels, 2012 [60]	1	0 (0)	0		0	0 (0)	0	0
Soccer fields	Cardon, 2008 57]	1	0 (0)	0	Haug, 2010^♂^[53]	6	2 (33)	0	0
Areas with markings					Willenberg, 2010 [51]				
					Haug, 2008 [54]				
					Sallis, 2001 [44]				
					Haug, 2010 [53]				
					Ridgers, 2010 [30,49]				
Structured track	Gubbels, 2012 [60]	1	1 (100)	+++		0	0 (0)	0	+++
Swinging equipment	Gubbels, 2012 [60]	1	1 (100)	- - -		0	0 (0)	0	- - -
Tunnels	Gubbels, 2012 [60]	1	0 (0)	0		0	0 (0)	0	0
Trash cans		0	0 (0)	0	Colabianchi, 2011 [40,46]	1	1 (100)	- - -	- - -
Water and swimming facilities		0	0 (0)	0	Haug, 2008 [54]	1	1 (100)	- -	- -
**ORGWARE**									
Decreased playground density (m^2^/child)	Cardon, 2008^♂^[57]	1	1 (100)	++	Ridgers, 2010 [30,49]	1	1 (100)	++	++
Increased recess duration	Cardon, 2008 [57]	1	1 (100)	++	Ridgers, 2010 [30,49]	1	0 (0)	0	?
No organized activities		0	0 (0)	0	McKenzie, 2010^♀^[48]	1	1 (100)	++	++
Supervision	Cardon, 2008^♀^[57]	1	1 (100)	- -	McKenzie, 2010 [48]	6	2 (33)	0	?
					McKenzie, 2010^♂^[48]				
					Willenberg, 2010 [51]				
					Sallis, 2001 [44]				
					Zask, 2001 [52]				
					Ridgers, 2010 [30,49]				

Studies with a + symbol indicate a significant positive association, studies with a - symbol indicate a significant negative association, and studies with a 0 symbol indicate no significant association; ♀/♂ in superscript indicates an association that only accounts for girls/boys. Summary codes: a 0 symbol as summary code indicates no association; a ? symbol as summary code indicates indeterminate/inconclusive association; a + symbol as summary code indicates a positive association and a – symbol as summary code indicates a negative association. The number of characters relate to the quality of the studies; one + or – for studies of poor quality (≤5.0 points), ++ or - for studies of moderate quality (5.5-8.0 points), and +++ or - - - for studies of high quality (≥8.5 points). Note: if ≥ 4 studies an additional + or – was also assigned [13].

In primary, secondary or mixed schools, summary codes of association indicate no associations of hardware playground characteristics with physical activity. Of the software characteristics, the provision of play equipment (++), balls (+++), portable (++) and fixed equipment (+++), and hopscotch/skipping rope area (+++) were positively associated with physical levels. With regard to the orgware playground characteristics, no organized activities (++) and decreased playground density (++) were positively associated with recess activities. Negative associations were found with physical activity for trash cans (- - -) and water and swimming facilities (- -).

When the associations between playground characteristics and physical activity are summarized regardless of school type (preschool or primary, secondary, or mixed school), there is no longer an association between hardware playground characteristics and children’s physical activity level. Decreased playground density and no organized activities are positively associated with children’s physical activity, as well as the provision of portable and fixed play equipment (including balls), the presence of a hopscotch/skipping rope area, an open field with no markings, and a structured track.

## Discussion

Overall, this review found moderate evidence for an effect of the provision of playground equipment on physical activity levels of children at preschools and primary, secondary or mixed schools. There was inconclusive evidence for an effect of the allocation of playground markings and more play space and for multi-component interventions on children’s health in terms of physical activity, cognitive and social outcomes. These results are in accordance with previous reviews that also showed no conclusive evidence of playground interventions at primary schools [22,24,25]. Evidence for playground intervention effects was only slightly different for primary schools and preschools. At primary schools, there was moderate evidence for an effect of the use of multi-component interventions on children’s physical activity level, in contrast to inconclusive evidence at preschools. For the other intervention strategies no (conclusive) evidence was found nor in primary schools nor in preschools.

### Boys versus girls

Four of the 13 experimental studies performed subgroup analyses and found stronger effects on the physical activity level and on post-recess attention of girls compared to boys. According to Verstraete et al. [35] this might be explained by the fact that boys are already very active at baseline level, making it difficult to find significant improvements due to an intervention. The fact that girls more often engage in social talk on playgrounds and choose sedentary play activities and games also make them more susceptible for improvements [49]. Lastly, it is known that boys often engage in ball games. Decreasing playground density, as performed in the study of Van Cauwenberghe et al. [42] may allow girls to increasingly engage in their own type of games/physical activities, without being dominated by e.g., boys or supervisors. Since the main reasons for the difference in playground physical activity levels between girls and boys are not yet known, future studies on this topic should contain subgroup analyses according to sex in their design.

### Additional findings from the observational studies

Next to 13 experimental studies, 17 observational studies have been summarized in our review. The observational studies indicate that among preschoolers, mainly hardware and orgware playground characteristics (i.e., increased playground size, decreased playground density, and increased recess duration) are associated with an increase in physical activity level during recess. However, among children on primary, secondary or mixed schools, software characteristics (i.e., play equipment) are mainly positively associated with increased physical activity levels. It seems that for preschool children, having sufficient space to play and having optimal playground conditions (open field, no supervision, longer recess duration) may be sufficient to be physically active.

### Observational studies versus experimental studies

Although randomized controlled trials remain a prominent study design in clinical research, they depend on plausibility and adequacy arguments from observational studies to make hypotheses about causal relationships credible [61,62]. Overall, results from observational studies in our review indicate that particularly hardware and orgware playground characteristics (i.e. playground size, decreased playground density and increased recess duration) are associated with children’s physical activity levels at preschools. However, experimental studies found no evidence for this, mainly due to the small amount of studies that investigated the effects of these strategies. Second, observational studies also show that the provision of play equipment is associated with children’s physical activity levels, particularly in primary, secondary or mixed schools. Unfortunately, experimental studies were not able to generate conclusive evidence for the provision of play equipment at primary, secondary or mixed schools. However, regardless of school type, we found moderate evidence for the use of play equipment. Aiming for observational and experimental studies to be complementary, fellow researchers are advised to take results from observational studies into account when designing an experimental study and vice versa.

### Limitations and recommendations

Based on study design, sample size, methodological quality and intervention effect, nearly all intervention strategies did not reach moderate or strong levels of evidence. At first sight, when looking at the percentage of studies that found positive effects, some playground interventions seemed to have significant beneficial effects in this review. However, because of a lack of large randomized controlled trials with high methodological quality in this review, levels of evidence did not reach moderate or strong levels. We realize that the application of a randomized study design in experimental studies is hampered by the nature of environmental interventions, and by the context of the study. For example, some interventions were based on governmental funding aiming to improve environmental facilities of deprived schools, and for this reason no randomization procedure could be carried out. However, we strongly advise researchers to conduct more large RCTs investigating environmental interventions, in order to draw conclusions that are more valid. The effects of the improvement of e.g., organizational factors in (pre)school playgrounds could be investigated by allocating a number of preschools to either an intervention or a control condition.

A second limitation is that the outcomes of this review do mainly account for physical activity and can therefore not be generalized to other types of outcomes. Investigation of the association of (pre)school playgrounds with cognitive, and social economic outcomes is highly relevant, and researchers are urgently invited to focus on these outcomes in future observational and experimental studies. With regard to generalizability, the results of this review are mainly limited to studies performed in preschools or primary schools. Only two studies explored associations of playground characteristics with physical activity during recess at secondary schools. Since many adolescents fail to achieve the requirements for sufficient physical activity [63], it is recommended to examine the value of playgrounds for this age group as well. Another limitation that should be kept in mind is the variability in the type of school playgrounds examined in this review. Although the majority of the studies were conducted in the United States or United Kingdom, playgrounds differ in e.g., their size, shape, vegetation, and climate, depending on their geographical location. These differences might have influenced the effects and associations found in the studies included in the review.

## Conclusion

Overall, findings demonstrate inconclusive evidence for positive effects of playground interventions in a (pre)school setting on children’s physical activity levels. Looking at the evidence on the value of different playground-based intervention strategies, moderate evidence was generated for an effect of the provision of play equipment at all school types, and for an effect of multi-component interventions, including the provision of playground markings, play equipment and/or play space on the physical activity level of children at primary schools. No evidence was found for other health effects of playground-based interventions in terms of cognitive and social outcomes. For preschool children, having sufficient time and space to play seems to be sufficient to be physically active. In primary, secondary or mixed schools on the other hand, the presence of fixed play equipment appears to be a predictor of children’s physical activity level during recess. In order to strengthen the findings of this review, researchers are advised to conduct more high quality experimental studies with a large sample size and randomized controlled design. Further, future research should examine the effect of playground-based interventions on other outcomes than physical activity. In addition, future research should also focused on additional assessment of implementation strategies and process evaluations to reveal which intervention strategies and playground characteristics are most effective.

## Competing interests

The authors declare that they have no competing interests.

## Authors’ contributions

SdV had the original idea for the study which she further developed with AMS. AMS conducted the literature search and selected the studies based on the title and the abstract together with KB. KB and AMS developed a review protocol that was used by all authors to extract the data. The study outcomes were summarized and reported by KB who drafted the manuscript. AMS and SdV helped to draft the manuscript. All authors read and approved the final manuscript.

## Supplementary Material

Additional file 1 Long-Search terms for the cross-database search in PubMed, PsycInfo and EMBASE. Click here for file

Additional file 2 **Table S1.** Methodological quality of experimental experimental (n = 13) and **Table S2.** Methodological quality of observational studies (n=17). Click here for file
