# Supplementary material for: The value of (pre)school playgrounds for children’s physical activity level: a systematic review
Source: Int J Behav Nutr Phys Act. 2014 May 3;11:59. doi: 10.1186/1479-5868-11-59 (PMC4031969; doi:10.1186/1479-5868-11-59)
Supplement: Additional file 1 — Long-Search terms for the cross-database search in PubMed, PsycInfo and EMBASE. [file 1479-5868-11-59-S1.docx]

**Appendix 1**

**Search terms for the cross-database search in PubMed, PsycInfo and EMBASE

PubMed** ((("schoolyard" OR "play and playthings"[MeSH Terms] OR ("play"[All Fields] AND "playthings"[All Fields]) OR "play and playthings"[All Fields] OR "play"[All Fields]) AND outdoor[All Fields]) OR playground[All Fields]) AND (("child"[MeSH Terms] OR "child"[All Fields]) OR ("adolescent"[MeSH Terms] OR "adolescent"[All Fields]) OR ("adolescent"[MeSH Terms] OR "adolescent"[All Fields] OR "youth"[All Fields]))

**PsycInfo**playgrounds OR outdoor play OR (outdoor and play)

**EMBASE**(((((({outdoor play} OR {outdoor activity} OR {outdoor activities} OR {playground facilities} OR {playground equipment} OR {free play} OR playtime OR {playground facility} OR {outdoor play} OR {free recess} OR {school break} OR { outdoor physical activity} OR { outdoor physical activities} OR { outdoor spaces} OR { schoolyard} OR {active playground}) AND (play OR playing)) AND ({social development} OR exercise OR {child development} OR effects OR importance OR benefits OR impact OR beneficial OR {psychological development} OR health OR lifestyle OR {active lifestyle} OR postive OR value OR values) AND PUBYEAR > 1999) AND (KEY(children) OR KEY(schoolchildren) OR KEY(child, preschool) OR KEY(toddlers) OR KEY(adolescents) OR INDEXTERMS(children) OR INDEXTERMS(child, preschool) OR INDEXTERMS(schoolchildren) OR INDEXTERMS(toddlers) OR INDEXTERMS(adolescents) OR KEY(preschool children) OR INDEXTERMS(preschool children) OR TITLE(children) OR TITLE(schoolchildren) OR TITLE(adolescents) OR TITLE(toddlers))) AND NOT (TITLE-ABS-KEY(exposure) OR TITLE-ABS-KEY(contamination) OR TITLE-ABS-KEY(contaminated) OR { soil contamination} OR { contaminated soil})) AND ({outdoor play} OR outdoor)) OR ({playground characteristics} OR { playground specifications} OR {type playground} OR playground PRE/3 TYPE OR {playground design}) AND (LIMIT-TO(SRCTYPE, "j")) AND (EXCLUDE(EXACTKEYWORD, "Injury") OR EXCLUDE(EXACTKEYWORD, "Wounds and Injuries") OR EXCLUDE(EXACTKEYWORD, "Accidental Falls") OR EXCLUDE(EXACTKEYWORD, "Environmental exposure"))
