# Supplementary material for: The value of (pre)school playgrounds for children’s physical activity level: a systematic review
Source: Int J Behav Nutr Phys Act. 2014 May 3;11:59. doi: 10.1186/1479-5868-11-59 (PMC4031969; doi:10.1186/1479-5868-11-59)
Supplement: Additional file 2 — Table S1. Methodological quality of experimental experimental (n = 13) and Table S2. Methodological quality of observational studies (n=17). [file 1479-5868-11-59-S2.pdf]

Additional file 1: Table S1: Methodological quality of experimental studies (n=13)

**First author**<br>**Year of publication**	Huberty<br>2011	Loucaides<br>2009	Ridgers<br>2007	Long-term<br>Ridgers<br>2007	Ridgers<br>2010	Stratton<br>2005	Stratton<br>2000	Holmes<br>2006	Van Cauwenberghe<br>2012	Bundy<br>2008	Cardon<br>2009	Colabianchi<br>2009	Brown, 2009<br>2009	Brink<br>2010	Hannon<br>2008	Verstraete<br>2006
**Methodological quality criteria**																
Are the individuals selected to participate in the study likely to be representative of the target population?	1	1	1	1	1	1	1	1	1	1	1	1	1	1	1	1
Is the age range specified?	0.5	0.5	1	0.5	0.5	1	1	1	1	1	1	0.5	1	1	1	0.5
Are inclusion and exclusion criteria of schools specified?	1	0	1	0	1	1	1	0	0	1	1	1	1	1	1	1
Is the response rate > 70%, or is the information on nonresponders sufficient to make inference on the representativeness of the study population?	1	0	n/a	n/a	n/a	n/a	0	0	0	0	1	1	0	0	1	0
Is the study period specified? (month *and* year)	1	1	1	1	1	0.5	0	0	1	0	1	1	1	0.5	1	0
Are important characteristics specified? (gender *and* SES)	0.5	0.5	0.5	0.5	0.5	0.5	0.5	0.5	0.5	0.5	0.5	1	0.5	1	0.5	0.5
Are the data collection tools reported to be valid? (regarding the measurement of outcome(s))	1	1	1	1	1	1	1	0	1	1	1	0	0	1	1	1
Are the data collection tools reported to be reliable? (regarding the measurement of outcome(s))	1	1	0	0	0	1	1	1	0	1	0	1	1	1	1	1
Is the statistical analytical procedure completely reported? (tests, subgroup analysis, statistical software package and P-value)	0.5	1	1	1	1	1	1	1	1	1	1	0.5	0	1	1	1
Is there a control group?	0	1	1	1	1	1	1	0	0	0	1	1	0	1	0	1
Are schools/participants being randomized?	0	1	0	0	0	0	0	0	0	0	1	0	0	0	0	1
Are all measures described that were used to blind study participants and personnel from knowledge of which intervention a partipant received? Is all information provided relating to whether the intended blinding was effective?	0	0	0	0	0	0	0	0	0	0	0	0	0	0	0	0
Are all measures decribed that were used to blind outcome assessors from knowledge of which intervention a participant received? Is all information provided relating to whether the intended blinding was effective?	0	0	0	0	0	0	0	0	0	0	0	0	0	0	0	0
Is the completeness of outcome data described for each main outcome, including attrition and exclusions from the analyses?	1	1	1	1	1	1	1	0	1	1	0.5	0.5	0	0	0.5	1
**Sum score (0-14)**	8.5	9	8.5	7	8	9	8.5	4.5	6.5	7.5	10	8.5	5.5	8.5	9	9

Items were derived from scoring lists of Prins et al. (2002) and De Vries et al. (2006) Each item was scored with ‘present/reported’ (1), ‘partly present/reported’ (0.5), or ‘absent/not reported’ (0), in accordance with De Vries et al. (2006).

Additional file 1: Table S2: Methodological quality of observational studies (n=17)

**First author**<br>**Year of publication**<br>**Methodological quality criteria**	Colabianchi 2011	Haug 2010	McKenzie 2010	Taylor 2011	Willenberg 2010	Nielsen 2010	Zask 2001	Nielsen 2012	Ridgers 2010	Fairclough 2011	Gubbels 2012	Cardon 2008	Haug 2008	Dowda 2009	Boldemann 2006	Brown 2009	Sallis 2001
Are the individuals selected to participate in the study likely to be representative of the target population?	1	1	1	1	1	1	1	1	1	1	1	1	1	1	1	1	1
Is the age range specified?	0	1	0	1	1	1	1	1	1	1	1	1	1	1	1	1	0.5
Are inclusion and exclusion criteria of schools specified?	1	1	1	1	1	1	1	1	0	1	1	1	1	1	1	1	0
Is the response rate > 70%, or is the information on nonresponders sufficient to make inference on the representativeness of the study population?	1	1	1	1	0	1	0	1	0	1	1	1	1	0	1	1	0
Is the study period specified? (month *and* year)	1	1	1	1	1	1	1	0.5	1	1	1	1	1	0	1	0.5	1
Are important characteristics specified? (gender *and* SES)	1	0.5	0.5	0.5	0.5	1	0.5	0.5	0.5	1	0.5	0.5	1	1	1	1	0.5
Are the data collection tools reported to be valid? (regarding the measurement of the independent variable)	0	0	0	0	0	0	1	0	0	0	0	0	0	0	0	0	0
Are the data collection tools reported to be reliable? (regarding the measurement of the independent variable)	1	0	0	1	0	0	1	0	0	0	1	0	0	0	0	1	0
Are the data collection tools reported to be valid? (regarding the measurement of outcome(s))	1	1	1	0	0	1	1	1	1	1	0	1	0	1	1	0	1
Are the data collection tools reported to be reliable? (regarding the measurement of outcome(s))	1	1	1	0	0	1	1	1	1	1	1	0	1	0	1	1	1
Is the statistical analytical procedure completely reported? (tests, subgroup analysis, statistical software package and P-value)	1	1	1	1	1	1	1	0.5	1	1	1	1	1	0	1	0.5	0.5
**Sum score (0-11)**	9	8.5	7.5	7.5	5.5	9	9.5	7.5	6.5	9	8.5	7.5	8	5	9	8	5.5

Items were derived from scoring lists of Prins et al. (2002) and De Vries et al. (2006) Each item was scored with ‘present/reported’ (1), ‘partly present/reported’ (0.5), or ‘absent/not reported’ (0), in accordance with De Vries et al. (2006).
